# Pre-existing iron deficiency anemia and long-term risk of recurrent acute kidney injury in survivors of ICU-associated AKI: a propensity-matched study

**DOI:** 10.3389/fnut.2026.1779193

**Published:** 2026-05-05

**Authors:** Kuo-Chuan Hung, Hsiu-Lan Weng, Chun-Ning Ho, Li-Kai Wang, Yi-Chen Lai, Jheng-Yan Wu, I-Wen Chen

**Affiliations:** 1 Department of Anesthesiology, Chi Mei Medical Center, Tainan, Taiwan; 2 School of Medicine, College of Medicine, National Sun Yat-sen University, Kaohsiung, Taiwan; 3 Department of Anesthesiology, E-Da Hospital, I-Shou University, Kaohsiung, Taiwan; 4 Department of Nutrition, Chi Mei Medical Center, Tainan, Taiwan; 5 Department of Anesthesiology, Chi Mei Medical Center, Liouying, Tainan, Taiwan

**Keywords:** acute kidney injury, chronic kidney disease, critical care, iron deficiency anemia, propensity score matching, recurrent acute kidney injury

## Abstract

**Background:**

Recurrent acute kidney injury (rAKI) is a common complication among critically ill patients and is associated with progressive renal deterioration and increased long-term morbidity. Iron deficiency anemia (IDA) may compromise renal adaptive responses through impaired oxygen delivery and cellular energetics; however, whether pre-existing IDA is associated with rAKI risk remains unclear.

**Methods:**

We conducted a propensity score-matched retrospective cohort study using the TriNetX database (2010–2024). Adults with AKI during intensive care unit admission were stratified by pre-existing IDA status diagnosed within 6 months before the index AKI episode. A 90-day landmark approach was employed to minimize immortal-time bias. The primary outcome was rAKI occurring between 3 months and 3 years. Secondary outcomes included progression to end-stage renal disease (ESRD), all-cause mortality, major adverse cardiovascular events (MACEs), and estimated glomerular filtration rate (eGFR) decline below 30 ml/min/1.73 m^2^.

**Results:**

After matching, 13,002 individuals were included in each cohort. Pre-existing IDA was associated with a significantly higher risk of rAKI (hazard ratio [HR], 1.53; 95% confidence interval [CI], 1.44–1.63; *p* < 0.001) and eGFR decline below 30 ml/min/1.73 m^2^ (HR, 1.19; 95% CI, 1.11–1.29; *p* < 0.001). These associations between IDA and rAKI risk persisted during the extended follow-up (3–5 years) and remained consistent across the sensitivity analyses and demographic subgroups. Although MACEs showed a statistically significant association (HR, 1.16; *p* < 0.001), the effect size was limited. The associations of IDA with long-term risks of ESRD or mortality were not observed.

**Conclusion:**

Pre-existing IDA is associated with an increased risk of rAKI and accelerated renal function decline among critically ill AKI survivors. However, owing to the observational design, a causal relationship cannot be established. Prospective investigations are warranted to validate these observations and to clarify the underlying biological mechanisms.

## 1 Introduction

Acute kidney injury (AKI) is a frequent and serious complication among critically ill patients in the intensive care unit (ICU), with reported incidence rates ranging from 20% to over 50%, depending on diagnostic criteria and patient populations (1, 2). AKI is characterized by an abrupt decline in renal function and is associated with adverse short- and long-term outcomes, including prolonged hospitalization, increased healthcare costs, cardiovascular morbidity, progression to chronic kidney disease (CKD), and increased mortality (3, 4). Among AKI survivors, recurrent AKI (rAKI) represents an important clinical concern, occurring in approximately 25%−30% of patients within several years of recovery (5–7). Recurrent episodes of rAKI are associated with progressively impaired renal recovery, which may cumulatively exacerbate kidney damage and contribute to both accelerated progression to CKD and increased long-term mortality (5, 8, 9). Therefore, identifying the factors that predispose patients to rAKI is essential for improving long-term renal outcomes.

Iron deficiency anemia (IDA) is among the most common nutritional deficiencies worldwide, affecting hundreds of millions of individuals and representing an enduring global public health burden (10, 11). Beyond its hematologic consequences, pre-existing IDA may increase susceptibility to organ injury through several biological mechanisms. Chronic anemia reduces systemic oxygen delivery, which may be detrimental to metabolically active tissues such as renal tubular epithelium (12, 13). Consistently, pre-existing anemia has also been associated with adverse renal outcomes following acute illnesses and surgical procedures (14–17). In addition, iron deficiency, independent of hemoglobin levels, has been associated with impaired cellular energetics, mitochondrial dysfunction, and increased oxidative stress (18–20). Through dysregulated hypoxia-inducible factor signaling and heightened oxidative stress, iron deficiency may further compromise renal adaptive responses to hypoxia and promote tubular injury, as reflected by elevated urinary biomarkers of tubular damage in patients with IDA (21–25).

Nevertheless, whether IDA preceding an index AKI episode is associated with an increased risk of rAKI in critically ill patients remains unclear. Therefore, this study aimed to investigate the association between pre-existing IDA and the risk of rAKI among critically ill AKI survivors. We hypothesized that antecedent IDA is associated with an increased risk of rAKI during long-term follow-up.

## 2 Methods

### 2.1 Study design and data source

We performed a retrospective matched cohort analysis using the TriNetX Global Collaborative Network. This federated research infrastructure integrates de-identified electronic health records from 169 healthcare institutions across multiple countries, encompassing demographic characteristics, diagnostic codes, procedural records, pharmaceutical data, and laboratory measurements from both academic medical centers and community hospitals. The federated architecture of the platform facilitates large-scale queries across participating sites while safeguarding patient confidentiality through aggregated data output. This database has been widely used in epidemiological and outcomes research across various medical fields (26–28). The analysis included records from January 2010 to December 2024. Ethical approval was obtained from the Institutional Review Board of Chi Mei Medical Center (No.11403-E01), with informed consent waived due to the retrospective nature and de-identification of the data.

### 2.2 Study population

This study identified adults aged ≥ 18 years who experienced AKI during their ICU stay. The index date corresponded to the documented AKI diagnosis within the critical care environment, which was identified using established clinical diagnostic codes. Participants were stratified into two groups according to their pre-existing IDA status. The exposed cohort included individuals with a confirmed IDA diagnosis within the 6-month window before their AKI episode, whereas the comparator cohort comprised individuals without any documented IDA during this antecedent period. This 6-month interval was selected because patients with IDA diagnosed in the distant past may have received treatment and achieved resolution of their anemia. Therefore, restricting the exposure window to 6 months increases the likelihood that identified patients had active IDA proximate to the index AKI episode.

Several exclusion criteria were applied to reduce confounding. Patients with pre-existing advanced nephropathy (CKD stages 4 or 5, end-stage renal disease, chronic dialysis dependence, or prior renal transplantation) were excluded, as were those requiring renal replacement therapy during the index hospitalization. To isolate the impact of IDA, individuals with other anemia etiologies, including vitamin B12 or folate deficiency and anemia of unspecified cause, were also excluded. Patients receiving dexmedetomidine during the ICU stay were removed from the analysis given its reported renoprotective effects (29). Finally, patients who developed rAKI during the 90-day landmark interval were excluded to ensure all participants remained at risk for incident recurrence throughout the observation period. A 90-day landmark approach was adopted to minimize immortal-time bias and reverse causation; consequently, only individuals surviving beyond this threshold were retained for outcome evaluation.

### 2.3 Propensity score matching

Propensity scores were generated using multivariable logistic regression models incorporating baseline covariates documented within the 3-year period preceding the index date. Variables included in the matching algorithm included demographic attributes (age, sex, and ethnicity), relevant comorbid conditions, and the most recent laboratory values prior to the index date (Supplementary Table 1). To account for the potential etiologies of AKI, the presence of respiratory failure or sepsis before the index date was incorporated into the matching process. Iron supplementation use was also balanced between the cohorts. Matching was executed using a 1:1 greedy nearest-neighbor algorithm without replacement, applying a caliper of 0.1 standard deviations of the logit-transformed propensity score. Balance between groups was evaluated using standardized mean differences, with values under 0.1 considered indicative of satisfactory equilibrium. The propensity score distribution overlap was visually assessed through density plot inspection.

### 2.4 Outcome assessment

The primary endpoint was rAKI that developed between 3 months and 3 years after the index date. Secondary endpoints encompassed progression to end-stage renal disease (ascertained via diagnostic codes and documentation of hemodialysis dependence), all-cause mortality, reduction in estimated glomerular filtration rate (eGFR) below 30 ml/min/1.73 m^2^, major adverse cardiovascular events (comprising cardiac death, myocardial infarction, and stroke), and thrombocytosis. Thrombocytosis was designated as a positive control outcome, given the well-documented relationship between IDA and reactive thrombocytosis. The follow-up period was initiated at the 90-day landmark and extended until outcome occurrence, death, final documented clinical contact, or study conclusion, whichever arose first. To evaluate the persistence of observed associations, outcomes were additionally examined across an extended observation window spanning 3–5 years.

### 2.5 Sensitivity and subgroup analyses

The robustness of the findings was evaluated using three sensitivity analyses. Model I was confined to patients with documented healthcare encounters between 3 months and 3 years to address potential ascertainment bias related to differential follow-up intensity. Model II was limited to data from 2018 to 2023 to capture contemporary clinical management patterns. Model III was restricted to patients without antecedent CKD to assess the associations among individuals with preserved baseline kidney function. Pre-planned subgroup analyses were used to investigate potential effect modifications according to sex and age stratification (18–65 years vs. >65 years).

### 2.6 Statistical analysis

Continuous variables are expressed as means with standard deviations, while categorical variables are reported as frequencies with percentages. Incomplete covariate data were addressed through available-case analysis. Within the propensity-matched cohorts, time-to-event analyses were conducted using Cox proportional hazards models to obtain hazard ratios (HRs) and corresponding 95% confidence intervals (CIs). Death was treated as a censoring event in the cause-specific hazard models. This approach was selected because the study objective was etiological (i.e., examining the association between IDA and rAKI risk among those at risk), for which cause-specific hazard estimation is the recommended analytical framework. To identify independent predictors of rAKI, multivariable Cox proportional hazards regression was performed incorporating clinically relevant covariates, including demographic factors, comorbidities, and IDA status. The proportional hazards assumption was evaluated through the examination of Schoenfeld residuals. A two-sided *p*-value below 0.05 was considered statistically significant for the primary outcome. Bonferroni correction was applied to the secondary endpoints to adjust for multiple testing, establishing the significance threshold at *p* < 0.01. E-values were computed to quantify the magnitude of unmeasured confounding factors required to negate the observed primary outcome association. Statistical analyses were executed within the TriNetX platform using its integrated analytic framework.

## 3 Results

### 3.1 Patient selection and baseline characteristics

**Figure 1:**
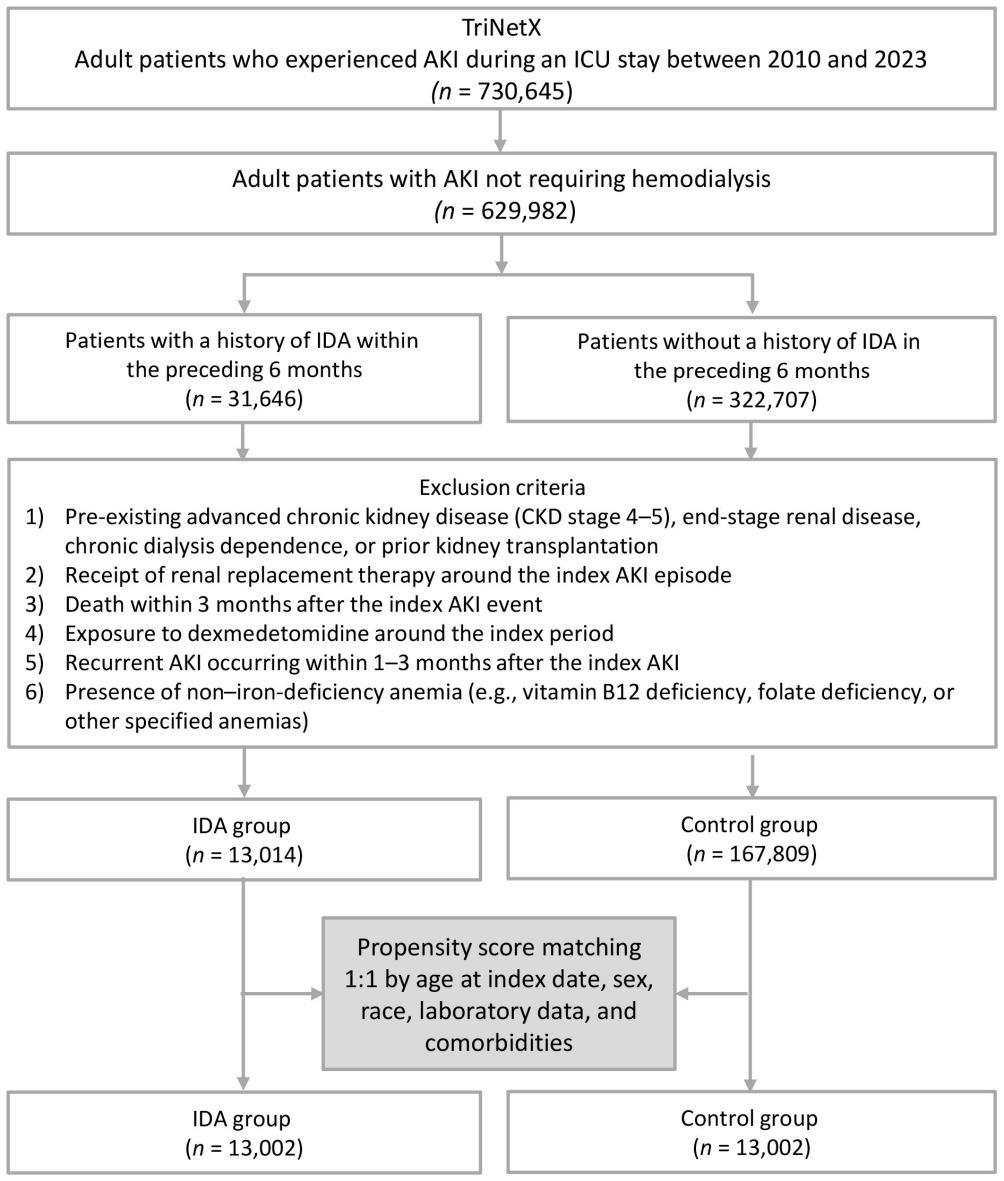
Patient selection flowchart. AKI, acute kidney injury; CKD, chronic kidney disease; IDA, iron deficiency anemia; ICU, intensive care unit.

**Figure 2:**
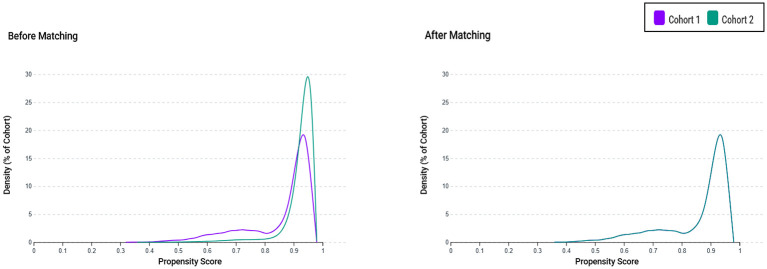
Propensity score distribution before and after matching. Density plots demonstrate the distribution of propensity scores in the iron deficiency anemia (IDA) group (Cohort 1) and the control group (Cohort 2) before (left panel) and after (right panel) propensity score matching. The substantial overlap between the two cohorts after matching supports the validity of the matching procedure.

A total of 13,014 critically ill patients with AKI and pre-existing IDA were initially identified from the TriNetX database, along with 167,809 patients without IDA. After matching, 13,002 individuals remained in each cohort for the analysis (Figure 1). Prior to matching, there were notable imbalances between the two groups. Patients with IDA were more likely to be female and exhibited a higher prevalence of multiple comorbidities, such as diabetes mellitus, ischemic heart disease, heart failure, respiratory failure, sepsis, and CKD. Laboratory parameters also differed between the groups, with the IDA cohort demonstrating higher proportions of patients with hypoalbuminemia, elevated C-reactive protein levels, and reduced eGFR. Iron supplementation was substantially more frequent in the IDA group. After matching, all baseline covariates achieved an adequate balance between the groups (Table 1). Density plots confirmed a substantial overlap in propensity score distributions between cohorts, supporting the validity of the matching procedure (Figure 2).

**Table 1:** Baseline characteristics of critically ill patients with acute kidney injury according to pre-existing iron deficiency anemia status before and after propensity score matching.

Variables	Before matching			After matching		
	IDA group (*n* = 13,014)	Control group (*n* = 167,809)	SMD	IDA group (*n* = 13,002)	Control group (*n* = 13,002)	SMD
Patient characteristics						
Age at index (years)	64.0 ± 17.1	61.3 ± 18.5	0.153	64.0 ± 17.1	64.3 ± 17.5	0.019
Female	6,559 (50.4)	63,682 (37.9)	0.253	6,547 (50.4)	6,678 (51.4)	0.020
BMI ≥35 kg/m^2^	2,724 (20.9)	31,686 (18.9)	0.051	2,722 (20.9)	2,692 (20.7)	0.006
White	7,986 (61.4)	111,368 (66.4)	0.104	7,983 (61.4)	7,989 (61.4)	0.001
Black or African American	3,484 (26.8)	35,408 (21.1)	0.133	3,476 (26.7)	3,452 (26.6)	0.004
Asian	444 (3.4)	5,745 (3.4)	0.001	444 (3.4)	443 (3.4)	< 0.001
Other Race	431 (3.3)	5,281 (3.1)	0.009	430 (3.3)	444 (3.4)	0.006
Comorbidities						
Essential (primary) hypertension	8,605 (66.1)	103,193 (61.5)	0.096	8,597 (66.1)	8,593 (66.1)	0.001
Diabetes mellitus	5,895 (45.3)	66,717 (39.8)	0.112	5,887 (45.3)	5,812 (44.7)	0.012
Ischemic heart diseases	5,759 (44.3)	62,345 (37.2)	0.145	5,753 (44.2)	5,760 (44.3)	0.001
Respiratory failure	5,441 (41.8)	60,679 (36.2)	0.116	5,434 (41.8)	5,456 (42.0)	0.003
Sepsis	5,205 (40.0)	50,000 (29.8)	0.215	5,195 (40.0)	5,232 (40.2)	0.006
Heart failure	5,155 (39.6)	47,065 (28.0)	0.246	5,144 (39.6)	5,153 (39.6)	0.001
Chronic kidney disease	4,522 (34.7)	41,355 (24.6)	0.222	4,511 (34.7)	4,483 (34.5)	0.005
Neoplasms	3,661 (28.1)	32,791 (19.5)	0.203	3,652 (28.1)	3,651 (28.1)	< 0.001
Severe sepsis	3,576 (27.5)	32,754 (19.5)	0.189	3,567 (27.4)	3,593 (27.6)	0.004
Nicotine dependence	3,100 (23.8)	41,048 (24.5)	0.015	3,097 (23.8)	3,016 (23.2)	0.015
Cerebrovascular diseases	2,879 (22.1)	34,961 (20.8)	0.031	2,879 (22.1)	2,855 (22.0)	0.004
COPD	2,822 (21.7)	28,496 (17.0)	0.119	2,819 (21.7)	2,761 (21.2)	0.011
Diseases of liver	2,591 (19.9)	23,601 (14.1)	0.156	2,581 (19.9)	2,622 (20.2)	0.008
Obstructive sleep apnea	2,033 (15.6)	21,348 (12.7)	0.083	2,029 (15.6)	1,954 (15.0)	0.016
Alcohol related disorders	1,456 (11.2)	18,536 (11.0)	0.005	1,455 (11.2)	1,526 (11.7)	0.017
Unspecified dementia	1,057 (8.1)	10,807 (6.4)	0.065	1,057 (8.1)	1,063 (8.2)	0.002
COVID-19	1,032 (7.9)	11,625 (6.9)	0.038	1,032 (7.9)	1,063 (8.2)	0.009
Long term (current) use of steroids	976 (7.5)	8,224 (4.9)	0.108	974 (7.5)	909 (7.0)	0.019
Laboratory data						
Albumin g/dl (< 3.5 g/dl)	8,089 (62.2)	75,218 (44.8)	0.353	8,078 (62.1)	8,195 (63.0)	0.019
eGFR < 60 ml/min/1.73m2	10,846 (83.3)	134,809 (80.3)	0.078	10,835 (83.3)	10,832 (83.3)	0.001
eGFR ml/min/1.73m2	45.9 ± 27.1	46.8 ± 24.9	0.035	45.9 ± 27.1	45.8 ± 26.7	0.004
Serum creatinine (mg/dl)	1.71 ± 1.29	1.68 ± 1.91	0.021	1.71 ± 1.29	1.69 ± 1.81	0.015
Hemoglobin A1c ≥9%	1,230 (9.5)	16,646 (9.9)	0.016	1,229 (9.5)	1,183 (9.1)	0.012
C-reactive protein ≥10 mg/L	2,066 (15.9)	17,591 (10.5)	0.160	2,061 (15.9)	2,036 (15.7)	0.005
Medication						
Insulins and analogs	5,511 (42.3)	65,281 (38.9)	0.070	5,507 (42.4)	5,426 (41.7)	0.013
Iron supplement	3,373 (25.9)	8,662 (5.2)	0.598	3,361 (25.9)	3,295 (25.3)	0.012
ACE inhibitors	3,357 (25.8)	37,380 (22.3)	0.082	3,357 (25.8)	3,319 (25.5)	0.007
Angiotensin II inhibitor	2,138 (16.4)	22,088 (13.2)	0.092	2,135 (16.4)	2,145 (16.5)	0.002
Biguanides	1,702 (13.1)	16,989 (10.1)	0.092	1,699 (13.1)	1,616 (12.4)	0.019
SGLT2 inhibitors	409 (3.1)	3,976 (2.4)	0.047	409 (3.1)	381 (2.9)	0.013
DPP-4 inhibitors	395 (3.0)	3,531 (2.1)	0.059	393 (3.0)	386 (3.0)	0.003
GLP-1 analogs	352 (2.7)	3,394 (2.0)	0.045	352 (2.7)	316 (2.4)	0.018

Data are presented as mean ± standard deviation for continuous variables and n (%) for categorical variables. IDA, iron deficiency anemia; BMI, body mass index; SMD, standardized mean difference; COPD, chronic obstructive pulmonary disease; eGFR, estimated glomerular filtration rate; GLP-1, glucagon-like peptide-1; SGLT2, sodium-glucose co-transporter 2; DPP-4, dipeptidyl peptidase 4; ACE, angiotensin-converting enzyme. SMD values < 0.1 indicate adequate balance between groups after matching.

### 3.2 Association between pre-existing IDA and outcomes

**Figure 3:**
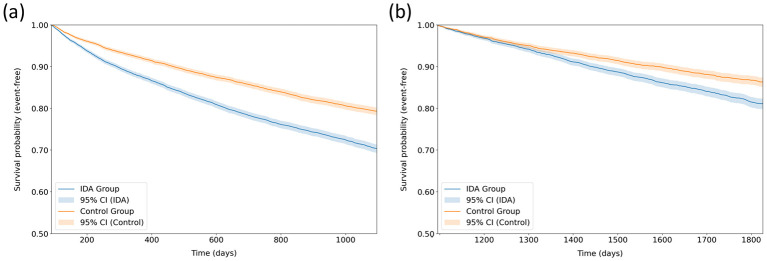
Kaplan-Meier curves for event-free survival from recurrent acute kidney injury according to pre-existing iron deficiency anemia status. **(a)** displays the survival probability (event-free) comparing the iron deficiency anemia (IDA) group vs. the control group during the primary follow-up period from 3 months to 3 years after the index date. **(b)** displays the survival probability (event-free) during the extended follow-up period from 3 to 5 years after the index date. The shaded areas represent 95% confidence intervals. IDA, iron deficiency anemia; CI, confidence interval.

During the 3-year follow-up period, pre-existing IDA was associated with a significantly higher risk of rAKI compared with the control group (HR, 1.53; 95% CI, 1.44–1.63; *P* < 0.001) (Table 2) (Figure 3a). The calculated E-value was 2.43 for the estimated effect and 2.24 for the lower confidence limit, indicating that a confounder would need to be moderately strongly associated with both the exposure and the outcome to fully explain the observed association. After Bonferroni correction, IDA was also associated with an increased risk of eGFR decline below 30 ml/min/1.73 m^2^ (HR, 1.19; 95% CI, 1.11–1.29; *P* < 0.001). Although MACE occurred more frequently in the IDA group (HR, 1.16; 95% CI, 1.08–1.24; *P* < 0.001), the observed association was of limited clinical magnitude. No significant associations were observed between IDA and all-cause mortality (*P* = 0.263) or progression to ESRD (*P* = 0.716). As a positive control outcome, thrombocytosis occurred more frequently in the IDA group (HR, 1.74; 95% CI, 1.32–2.30; *P* < 0.001), confirming the expected biological relationship.

**Table 2:** Association between pre-existing iron deficiency anemia and clinical outcomes during 3-year follow-up in propensity score-matched cohorts.

Outcomes	IDA group^‡^ events (%)	Control group^‡^ events (%)	HR (95% CI)	*P*-value	Significant after adjust
rAKI	2,442 (18.8%)	1,632 (12.6%)	1.53 (1.44, 1.63)	<0.001	NA
ESRD	93 (0.7%)	96 (0.7%)	0.95 (0.71, 1.26)	0.716	NS
Mortality	1,593 (12.3%)	1,499 (11.5%)	1.04 (0.97, 1.12)	0.263	NS
MACEs	1,699 (13.1%)	1,451 (11.2%)	1.16 (1.08, 1.24)	<0.001	S
eGFR < 30 ml/min/1.73m^2^	1,485 (11.4%)	1,235 (9.5%)	1.19 (1.11, 1.29)	<0.001	S
Thrombocytosis	137 (1.1%)	77 (0.6%)	1.74 (1.32, 2.30)	<0.001	S

^‡^N = 13,002 for each group. IDA, iron deficiency anemia; HR, hazard ratio; CI, confidence interval; rAKI, recurrent acute kidney injury; ESRD, end-stage renal disease; MACEs, major adverse cardiovascular events; eGFR, estimated glomerular filtration rate. Bonferroni correction was applied to the secondary outcomes to adjust for multiple comparisons (significance threshold: P < 0.01). S, significant after correction; NS, not significant after correction; NA, not applicable (primary outcome).

During the extended follow-up period (3–5 years), the association between IDA and rAKI persisted (HR, 1.39; 95% CI, 1.24–1.55; *P* < 0.001) (Figure 3b), while associations with mortality, ESRD, and MACE were non-significant (Table 3). The positive control outcome of thrombocytosis continued to demonstrate a strong association with IDA (HR, 2.97; 95% CI, 1.71–5.15; *P* < 0.001).

**Table 3:** Association between pre-existing iron deficiency anemia and clinical outcomes during extended follow-up (3–5 years) in propensity score-matched cohorts.

Outcomes	IDA group^‡^ events (%)	Control group^‡^ events (%)	HR (95% CI)	*P*-value
rAKI	726 (5.6%)	536 (4.1%)	1.39 (1.24–1.55)	<0.001
ESRD	53 (0.4%)	49 (0.4%)	1.09 (0.74–1.60)	0.674
Mortality	344 (2.6%)	336 (2.6%)	1.03 (0.89–1.20)	0.705
MACEs	487 (3.7%)	469 (3.6%)	1.05 (0.92–1.19)	0.469
eGFR < 30 ml/min/1.73m^2^	489 (3.8%)	416 (3.2%)	1.19 (1.04–1.35)	0.011
Thrombocytosis	50 (0.4%)	17 (0.1%)	2.97 (1.71–5.15)	<0.001

^‡^N = 13,002 for each group. IDA, iron deficiency anemia; HR, hazard ratio; CI, confidence interval; rAKI, recurrent acute kidney injury; ESRD, end-stage renal disease; MACEs, major adverse cardiovascular events; eGFR, estimated glomerular filtration rate. Hazard ratios were estimated using Cox proportional hazards models.

### 3.3 Sensitivity analyses

The association between IDA and rAKI was consistent across all sensitivity analyses (Table 4). In analyses restricted to patients with documented follow-up encounters (Model I), IDA was associated with a 47% higher risk of rAKI (HR, 1.47; *p* < 0.001). Comparable estimates were observed in the contemporary cohort from 2018 to 2023 (Model II; HR, 1.46; *p* < 0.001) and among patients without pre-existing CKD (Model III; HR, 1.48; *p* < 0.001). Associations with eGFR decline to < 30 ml/min/1.73 m^2^ demonstrated similar stability across all models.

**Table 4:** Sensitivity analyses for the association between pre-existing iron deficiency anemia and clinical outcomes during 3-year follow-up.

Outcomes	Model I		Model II		Model III	
	HR (95% CI)	*p*-value	HR (95% CI)	*p*-value	HR (95% CI)	*p*-value
rAKI	1.47 (1.38–1.56)	<0.001	1.46 (1.36–1.57)	<0.001	1.48 (1.35–1.62)	<0.001
ESRD	1.00 (0.75–1.34)	0.980	1.15 (0.82–1.60)	0.417	1.02 (0.64–1.62)	0.944
Mortality	1.03 (0.96–1.11)	0.433	1.01 (0.93–1.10)	0.802	1.06 (0.97–1.16)	0.209
MACEs	1.10 (1.03–1.18)	0.006	1.13 (1.05–1.22)	0.002	1.12 (1.02–1.22)	0.017
eGFR < 30 ml/min/1.73m^2^	1.20 (1.11–1.29)	<0.001	1.22 (1.12–1.33)	<0.001	1.15 (1.03–1.29)	0.017
Thrombocytosis	1.60 (1.22–2.10)	0.001	1.56 (1.19–2.05)	0.001	1.59 (1.15–2.21)	0.005

HR, hazard ratio; CI, confidence interval; rAKI, recurrent acute kidney injury; ESRD, end-stage renal disease; MACEs, major adverse cardiovascular events; eGFR, estimated glomerular filtration rate. Model I: restricted to patients with documented healthcare encounters between 3 months and 3 years (n = 10,014 per group). Model II was restricted to patients from 2018 to 2023 to reflect contemporary clinical practice (n = 9,772 per group). Model III: restricted to patients without antecedent chronic kidney disease (n = 9,042 per group). Hazard ratios were estimated using Cox proportional hazards models.

### 3.4 Subgroup analyses

When stratified by age, IDA was associated with a higher risk of rAKI in both younger and older patients, with comparable effect estimates across age groups (Table 5). A similar consistency was observed in sex-specific analyses, in which the association persisted among both males and females (Table 6). Overall, these results indicate that the relationship between IDA and rAKI is broadly consistent across key demographic subgroups.

**Table 5:** Subgroup analyses for the association between pre-existing iron deficiency anemia and clinical outcomes stratified by age category.

Outcomes	Age 18–65 years (*N*= 4,514 for each group)		Age >65 years (*N*= 8,466 for each group)	
	HR (95% CI)	*P*-value	HR (95% CI)	*P*-value
rAKI	1.55 (1.39–1.73)	<0.001	1.48 (1.37–1.60)	<0.001
ESRD	1.05 (0.67–1.67)	0.827	0.90 (0.62–1.31)	0.587
Mortality	1.05 (0.89–1.23)	0.574	0.99 (0.92–1.07)	0.878
MACEs	1.13 (0.99–1.29)	0.069	1.07 (0.99–1.16)	0.095
eGFR < 30 ml/min/1.73m^2^	1.08 (0.94–1.24)	0.286	1.18 (1.08–1.29)	<0.001
Thrombocytosis	1.89 (1.27–2.83)	0.002	1.41 (0.97–2.06)	0.071

HR, hazard ratio; CI, confidence interval; rAKI, recurrent acute kidney injury; ESRD, end-stage renal disease; MACEs, major adverse cardiovascular events; eGFR, estimated glomerular filtration rate.

**Table 6:** Subgroup analyses for the association between pre-existing iron deficiency anemia and clinical outcomes stratified by sex.

Outcomes	Male (*N*= 6,455 for each group)		Female (*N*= 6,544 for each group)	
	HR (95% CI)	*P*-value	HR (95% CI)	*P*-value
rAKI	1.64 (1.50–1.79)	<0.001	1.43 (1.31–1.56)	< 0.001
ESRD	0.84 (0.55–1.30)	0.437	1.20 (0.81–1.79)	0.360
Mortality	1.02 (0.92–1.12)	0.719	1.00 (0.90–1.10)	0.969
MACE	1.12 (1.02–1.24)	0.019	1.10 (0.99–1.21)	0.065
eGFR < 30 ml/min/1.73m^2^	1.26 (1.11–1.42)	<0.001	1.15 (1.05–1.27)	0.004
Thrombocytosis	2.15 (1.33–3.49)	0.001	1.73 (1.22–2.47)	0.002

HR, hazard ratio; CI, confidence interval; rAKI, recurrent acute kidney injury; ESRD, end-stage renal disease; MACEs, major adverse cardiovascular events; eGFR, estimated glomerular filtration rate.

### 3.5 Risk factors for recurrent AKI

Multivariate Cox proportional hazards regression identified several independent predictors of rAKI during the 3-year follow-up period (Table 7). After adjusting for demographic and clinical covariates, pre-existing IDA remained significantly associated with rAKI (HR, 1.58, *P* < 0.001). Among the comorbid conditions, CKD demonstrated the strongest association with rAKI (HR, 1.45, *P* < 0.001), followed by diabetes mellitus (HR, 1.35, *P* < 0.001) and heart failure (HR, 1.28, *P* < 0.001). Advancing age (HR per year, 1.01; *P* < 0.001), essential hypertension (HR, 1.16; *P* < 0.001), nicotine dependence (HR, 1.22; *P* < 0.001), chronic obstructive pulmonary disease (HR, 1.11; *P* < 0.001), and alcohol-related disorders (HR, 1.12; *P* < 0.001) showed mild but statistically significant associations. In contrast, male sex, ischemic heart disease, and being overweight or obese were not significantly associated with rAKI.

**Table 7:** Multivariable Cox proportional hazards regression for independent predictors of recurrent acute kidney injury during 3-year follow-up.

Variable	Hazard ratio (95% CI)	*P*-value
Pre-existing IDA (vs. no IDA)	1.58 (1.52–1.65)	<0.001
Male	1.00 (0.97–1.03)	0.930
Age at index (per year)	1.01 (1.00–1.01)	<0.001
Essential (primary) hypertension	1.16 (1.13–1.20)	<0.001
Diabetes mellitus	1.35 (1.32–1.39)	<0.001
Heart failure	1.28 (1.24–1.32)	<0.001
Chronic obstructive pulmonary disease	1.11 (1.07–1.14)	<0.001
Ischemic heart diseases	1.01 (0.98–1.04)	0.427
Chronic kidney disease	1.45 (1.41–1.49)	<0.001
Overweight and obesity	1.02 (0.99–1.06)	0.130
Nicotine dependence	1.22 (1.18–1.26)	<0.001
Alcohol-related disorders	1.12 (1.07–1.17)	<0.001

HR, hazard ratio; CI, confidence interval; IDA, iron deficiency anemia. Age was analyzed as a continuous variable (per 1-year increment).

## 4 Discussion

This propensity score-matched cohort study examined the relationship between pre-existing IDA and subsequent renal and cardiovascular outcomes among critically ill AKI survivors over both the primary (3-month to 3-year) and extended (3-year to 5-year) follow-up periods. A strong association between antecedent IDA and rAKI was evident and remained stable across both observation windows, various analytical methods, and all examined demographic subgroups. Among secondary outcomes, IDA was associated with accelerated decline in renal function as evidenced by progression to eGFR below 30 ml/min/1.73 m^2^, while associations with ESRD, all-cause mortality, and MACE were either non-significant or of limited clinical magnitude. These findings highlight IDA as a potentially modifiable factor that warrants clinical attention in patients recovering from critical illness-associated AKI.

The cumulative incidence of rAKI in our study was 18.8% in the IDA cohort and 12.6% in the controls over the 3-year follow-up period. Previous investigations have reported varying recurrence rates depending on population characteristics and follow-up duration. Holmes et al. observed that 29.3% of AKI survivors experienced a second episode in a large Welsh population-based cohort (5). Li et al. reported that 18.3% of elderly patients with early AKI recovery developed recurrent episodes during follow-up (30). Similarly, Altawalbeh et al. documented rAKI in approximately 20% of survivors within 5 years (31). The somewhat lower incidence in our study likely reflects our methodological approach, as patients who developed rAKI during the 90-day landmark period were excluded to ensure that all participants were genuinely at risk for incident recurrence.

To our knowledge, this is one of the first large-scale investigations to specifically examine the relationship between pre-existing IDA and rAKI among critically ill patients. Importantly, the observed association remained consistent across both the primary 3-year and extended 3–5-year follow-up periods, reinforcing the temporal stability of this relationship. Prior studies have identified diabetes mellitus, CKD, heart failure, and advanced age as established risk factors for rAKI (5, 6). Our multivariable analysis confirmed these associations, while demonstrating that IDA confers an additional independent risk. Our findings suggest that clinicians should recognize antecedent IDA as a potential risk factor and consider closer renal function monitoring in AKI survivors with pre-existing IDA.

In the current study, multiple analytical strategies were employed to evaluate the reliability of the observed associations. The *E*-value of 2.43 for the primary outcome indicates that an unmeasured confounder would need to be associated with both IDA exposure and rAKI by at least this magnitude to fully explain the observed relationship, suggesting reasonable robustness against residual confounding. The inclusion of thrombocytosis as a positive control outcome strengthens the confidence in our findings. The well-established biological relationship (32) between IDA and reactive thrombocytosis was consistently demonstrated throughout the observation period, confirming that our study methodology could detect known pathophysiological associations. This internal validation supports the credibility of the primary findings. Sensitivity analyses further reinforced the stability of the results. Restricting analyses to patients with documented healthcare encounters addressed potential ascertainment bias, while limiting them to contemporary data ensured their relevance to current clinical practice. The consistent effect estimates observed when excluding patients with pre-existing CKD suggest that the IDA-rAKI association exists independently of baseline nephropathy, strengthening the generalizability of the findings across varying degrees of baseline renal function.

The lack of significant associations between IDA and progression to ESRD or all-cause mortality merits further consideration. The relatively low event rate for ESRD (0.7% in both cohorts) likely provided insufficient statistical power to detect any significant differences. Additionally, the multifactorial nature of mortality in critically ill populations, encompassing cardiovascular, infectious, and malignant etiologies, may dilute the specific contribution of IDA to overall survival. Furthermore, the 90-day landmark design, while methodologically necessary to reduce immortal-time bias, excluded early deaths and may have been selected for survivors with inherently better prognoses, potentially attenuating mortality differences between the groups. Although a statistically significant association with MACEs was observed (HR, 1.16), the relatively small effect size suggests that the clinical impact may be limited. Propensity score matching balanced numerous cardiovascular-related comorbidities between cohorts, which may have attenuated the independent contribution of IDA to cardiovascular outcomes.

The significant association between IDA and progression to eGFR below 30 ml/min/1.73 m^2^ suggests that pre-existing IDA may be linked to accelerated nephron loss beyond recurrent acute episodes. This threshold represents an important clinical milestone, corresponding to CKD stage 4 and approaching the requirement for renal replacement therapy. The relationship between IDA and progressive decline in renal function may reflect cumulative tubular damage from repeated subclinical injuries. Iron deficiency has been associated with impaired regenerative capacity and maladaptive repair processes, potentially promoting fibrosis rather than functional recovery following kidney injury (33, 34). The persistence of this association during extended follow-up further supports a sustained rather than transient relationship. Notably, this finding has particular clinical relevance because eGFR decline represents a continuous pathological process that is amenable to intervention. Unlike binary endpoints such as ESRD requiring dialysis initiation, progressive eGFR reduction may identify patients at earlier stages when therapeutic strategies, including iron repletion, might still modify the disease trajectory. The consistent findings across sensitivity analyses reinforce the potential importance of addressing IDA in comprehensive kidney protection strategies.

While our findings suggest that pre-existing IDA is associated with adverse renal outcomes, whether iron supplementation should be administered to critically ill patients remains controversial. Iron is an essential nutrient for bacterial growth, and elevated free iron levels may theoretically promote pathogen proliferation and increase susceptibility to infection (35). Current evidence has raised concerns that intravenous iron administration during active critical illness could exacerbate infectious complications (36). Consequently, the optimal timing and route of iron repletion in this population requires careful consideration. Whether iron supplementation initiated after resolution of acute illness or during the post-ICU recovery phase might mitigate rAKI risk without augmenting the infection hazard warrants further investigation. Future studies should address these safety concerns while evaluating the potential renoprotective benefits of IDA correction in survivors of AKI.

Several limitations warrant acknowledgment. First, given the retrospective observational nature of the study, causal relationships cannot be definitively established, and the possibility of residual confounding from unmeasured factors persists despite rigorous propensity score matching and E-value analyses. Furthermore, death was handled as a censoring event rather than as a competing risk in the cause-specific hazard models. This approach is appropriate for etiologic analyses, although Kaplan–Meier–based cumulative incidence estimates may slightly overstate absolute event rates. Nonetheless, because mortality was well balanced between the cohorts and any remaining bias would likely be conservative in direction, competing risks are unlikely to have materially affected the observed hazard ratios. Second, IDA diagnosis relies on administrative coding, which may introduce misclassification bias; however, such non-differential misclassification would typically bias the results toward the null, suggesting that our estimates may be conservative. Third, the TriNetX database lacks granular data on IDA severity, iron indices, treatment adequacy, and specific AKI etiologies, which limits mechanistic interpretation. Additionally, the TriNetX database does not contain formal physiological severity scores (e.g., APACHE II or SOFA), and although our matching model incorporated respiratory failure, sepsis, and multiple comorbidity and laboratory proxies for illness severity, residual differences in the severity of the index critical illness between the two cohorts cannot be excluded. Fourth, the outcomes were identified using diagnostic codes without adjudication, potentially affecting precision. Fifth, the predominantly US-based healthcare organization network may limit generalizability to other healthcare systems or populations with different anemia prevalence patterns. Sixth, although the 90-day landmark design reduced immortal-time bias, it excluded patients who died or experienced early recurrence, potentially introducing survivorship bias. Finally, information regarding iron supplementation compliance and response during follow-up was unavailable, precluding the assessment of treatment effects on outcomes.

## 5 Conclusion

Pre-existing IDA is independently associated with an increased risk of rAKI and accelerated renal function decline among critically ill AKI survivors. However, whether iron supplementation can mitigate these risks remains uncertain, particularly given the concerns regarding potential susceptibility to infection in critically ill populations. Future prospective studies should investigate the optimal timing and safety of iron repletion strategies, specifically addressing whether correction of IDA during the post-ICU recovery phase—rather than during active critical illness—can reduce the risk of recurrence without augmenting infectious complications. Randomized controlled trials are warranted to establish evidence-based recommendations for managing IDA in this vulnerable population.

## Data Availability

The raw data supporting the conclusions of this article will be made available by the authors, without undue reservation.
