# Supplementary material for: Pre-existing iron deficiency anemia and long-term risk of recurrent acute kidney injury in survivors of ICU-associated AKI: a propensity-matched study
Source: Front Nutr. 2026 May 5;13:1779193. doi: 10.3389/fnut.2026.1779193 (PMC13183569; doi:10.3389/fnut.2026.1779193)
Supplement: Supplementary file 1 [file Table_1.docx]

**Table S1. Definitions of cohort eligibility, matching variables, and outcomes (with codes)**

| Category | Item | Definition / Codes |
| --- | --- | --- |
| Inclusion criteria | Age | ≥ 18 years |
|  | Index AKI | Acute kidney failure: ICD-10-CM N17 |
|  | Critical illness | Critical Care Services: CPT 1013729 |
|  | Index period | Jan 1, 2010 – Dec 31, 2023 |
| Exposure definition | Iron deficiency anemia (IDA) | ICD-10-CM D50 |
| Exclusion criteria | Other anemias | D64 (other anemias), D51 (vitamin B12 deficiency anemia), D52 (folate deficiency anemia), D53 (other nutritional anemias) |
|  | Advanced CKD / ESRD | N18.4 (CKD stage 4), N18.5 (CKD stage 5), N18.6 (ESRD) |
|  | Dialysis dependence | Z99.2, ICD-10-PCS 5A1D70Z, ICD-10-PCS 5A1D |
|  | Kidney transplant | Z94.0 |
|  | Early mortality | Deceased status, R99 (ill-defined and unknown cause of mortality) |
|  | Dexmedetomidine exposure | RxNorm 48937 |
|  | Recurrent AKI near index | N17 within 1–3 months after index |
| Variables for matching (PSM) | Demographics | Age, sex, race |
|  | Cardiovascular | Hypertension (I10), ischemic heart disease (I20–I25), heart failure (I50), cerebrovascular disease (I60–I69) |
|  | Metabolic | Diabetes mellitus (E08–E13), obesity (BMI), dyslipidemia |
|  | Pulmonary | COPD (J44), respiratory failure (J96), obstructive sleep apnea (G47.33) |
|  | Renal | Chronic kidney disease (N18) |
|  | Infection | Sepsis (A41), severe sepsis (R65.2), COVID-19 (U07.1) |
|  | Neurologic | Dementia (F03) |
|  | Liver disease | K70–K77 |
|  | Substance use | Nicotine dependence (F17), alcohol-related disorders (F10) |
|  | Medications | Insulin (A10A), biguanides (A10BA), DPP-4 inhibitors (A10BH), GLP-1 agonists (A10BJ), SGLT-2 inhibitors (A10BK), ACE inhibitors (CV800), ARBs (CV805), iron supplementation (TN410) |
|  | Laboratory | Albumin (LOINC 9045), HbA1c (9037), eGFR (MDRD, TNX:8001), CRP (9063), BMI (9083) |
| Outcomes | AKI | ICD-10-CM N17 |
|  | ESRD | N18.6, Z99.2 |
|  | Mortality | Deceased status, R99 |
|  | MACE | Cardiac arrest (I46), acute myocardial infarction (I21), cerebral infarction (I63) |
|  | Sepsis | A41 |
|  | eGFR < 30 mL/min/1.73 m² | MDRD-based eGFR ≤ 30: TNX:8001 |
|  | Thrombocytosis | D75.83 |
